# Novel Expression of EGFL7 in Osteosarcoma and Sensitivity to Cisplatin

**DOI:** 10.3389/fonc.2020.00074

**Published:** 2020-02-06

**Authors:** Qing Liu, Hongbo He, Yuhao Yuan, Hao Zeng, Zhiwei Wang, Wei Luo

**Affiliations:** ^1^Department of Orthopaedics, Xiangya Hospital, Central South University, Changsha, China; ^2^Department of Spine Surgery, The Second Xiangya Hospital of Central South University, Changsha, China

**Keywords:** epidermal growth factor-like domain 7, osteosarcoma, angiogenesis, chemotherapy, therapeutic target

## Abstract

Epidermal growth factor-like domain 7 (EGFL7) is a protein specifically secreted by blood vessel endothelial cells, which plays an important role in angiogenesis. Considering the aberrant secretion of EGFL7 in osteosarcoma (OS) has not yet been elucidated, this study investigated the secretion of EGFL7 in OS and the changes in its secretion after chemotherapy. We observed increased varying secretion of EGFL7 in OS tissues compared with chondrosarcoma (CS) tissues. OS cell lines and HUVECs showed higher EGFL7 mRNA and protein expression than SW1353, with OS cells expressing the highest levels. In patient samples, EGFL7 was highly expressed in the cytoplasm of OS tumor cells and vascular endothelium cells. This overexpression was abolished in OS cell and tumor tissues when treated with chemotherapy. This study is a pioneering study to investigate EGFL7 expression and localization in human OS tissues and cell. Overexpression of EGFL-7 in response to chemotherapy suggests that it can be used as a therapeutic target for OS.

## Introduction

Osteosarcoma (OS), a tumor of mesenchymal origin, is the most common malignant primary bone tumor of adolescents and young adults, in which the annual incidence peaks at 8–11 million/year at 15–19 years of age. OS account for 15% of all solid extracranial cancers in this age group (1). Nearly 80% of OS patients are metastatic or micro-metastatic at diagnosis, which results in a poor prognosis due to metastatic relapse or recurrence (2). Multiagent and dose-intensive chemotherapy are the main treatment options, however, chemotherapy still relies on the same drugs as in the early 1980s and survival rates have not improved since then (1). In recent years, targeting therapy has been successfully applied to the treatment of various solid tumors and has achieved good results. However, poor options in the treatment of OS are due to a lack of specific tumor markers and therapeutic targets. In order to improve the clinical results of OS treatment, it is necessary to elucidate the biological mechanisms of this disease and identify targets for novel treatment modalities (1, 2).

Epidermal growth factor-like domain 7 (*EGFL7*), a 41-kDa secreted factor that is highly conserved in vertebrates, is a protein containing two epidermal growth factor-like (EGF-like) domains (3). *EGFL7* is unique because it is almost exclusively expressed by and acts on endothelial cells, playing an important role in facilitating angiogenesis in normal organs during development (4). Interestingly, *EGFL7* expression is high during embryonic and neonatal development, down-regulated in almost all mature tissues, and increases again during vascular injury or tumorigenesis (3). It has been demonstrated that *EGFL7* can regulate the collective migration of endothelial cells (ECs) and acts as a chemoattractant for cell migration to promote the angiogenesis of tumors (5), and tumor escape from immunity by repressing ECs activation (6). Recent studies revealed that *EGFL7* expressed and correlated with clinical features in several tumors, suggesting its potential use as a therapeutic target for cancers including breast cancer (7), epithelial ovarian cancer (8), hepatocellular carcinoma (9), colorectal cancer (10), acute myeloid leukemia (11), and malignant glioma (12, 13). Our previous study found a correlation between *EGFL7* expression with clinicopathological features of osteosarcoma (14). However, the expression and localization of *EGFL7* in human OS tissues and cells is still unclear and further research is needed.

The aim of this study is to investigate *EGFL7* mRNA and protein expression in OS cells and tissues, and its changes in expression after chemotherapy. *EGFL7* mRNA and protein expression was found to increase in OS cell lines and tumor tissues of OS. Both OS tumor cells and EC secreted *EGFL7* protein. After chemotherapy, *EGFL7* mRNA and protein expression decreased significantly, suggesting that *EGFL7* has the potential for use as a therapeutic target of OS.

## Materials and Methods

### Tissue Samples

**Figure 1:**
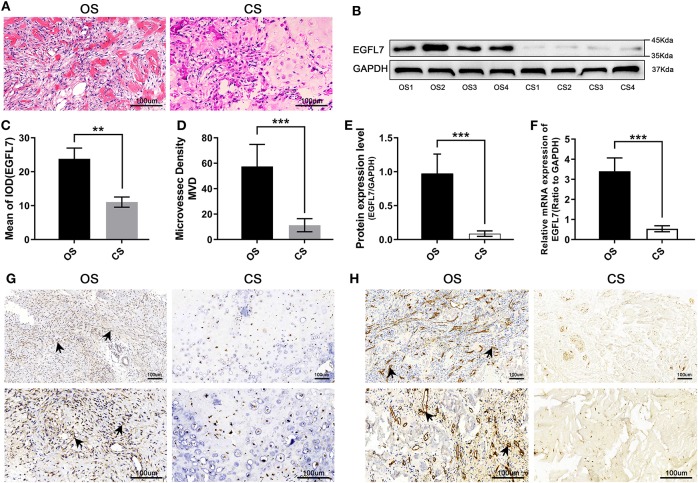
Compared with CS, EGFL7 is highly expressed in OS. **(A)** Typical pathological features of OS and CS revealed by HE staining, tumor-like osteogenesis can be seen in OS and cartilage-like matrix in CS. **(B,C)** Immunohistochemical of EGFL7 protein expression in OS tissue (*n* = 2) and CS controls (*n* = 2), comparison of IHC results from different magnification (100 and 200 ×) and its significance. High expression of EGFL7 (++) was found in OS tissues but negative in CS, the arrow refers to the positive area. The semi-quantitative statistical analysis based on IHC results shows that there is a significant difference in the expression of EGFL7 protein between OS and CS. **(D,E)** Western blot results showed that EGFL7 protein was overexpressed in OS tissue compared with CS, Student's *t*-test shows that the difference between OS and CS has obvious statistical significance. The abundance of EGFL7 protein in OS tissue was similar to that of GAPDH protein. **(F)** RT-PCR results showed that the transcription level of EGFL7 mRNA in OS tissues was significantly higher than that in CS tissues, and the difference was statistically significant. **(G)** Immunohistochemical results of chondrosarcoma and osteosarcoma showed that EGFL7 was highly expressed in osteosarcoma. **(H)** The immunohistochemical results of chondrosarcoma and osteosarcoma showed that CD34 was highly expressed in osteosarcoma, indicating that there were more abundant blood vessels in osteosarcoma. *P < 0.05, **P < 0.01, ***P < 0.001.

The study was approved by the Ethics Committee of Xiangya Hospital of Central South University, and informed consent was acquired from each patient. Twelve pairs of OS tumor tissues were acquired from OS patients who had undergone neo-adjuvant chemotherapy and resection surgery in Xiangya Hospital Bone Tumor Center. The untreated OS tumor tissues were obtained from OS patients who had undergone biopsy puncture pathology before neo-adjuvant chemotherapy (Figure 1A). The treated OS tumor tissues were obtained from resection surgery after neo-adjuvant chemotherapy, with visible enveloped residual tumor tissue. Neo-adjuvant chemotherapy included two cycles of adriamycin, cisplatin, and ifosfamide. Chondrosarcoma (CS) tumor tissues were obtained from CS patients who underwent segmental resection (Figure 1A). All tumors were identified by pathological examination. Tissues from the same patient were used as a matched pair. All tumor tissues were snap-frozen in liquid nitrogen and stored at −80°C until further use.

### Cell Lines

Human umbilical vein endothelial cells (HUVECs), human chondrosarcoma cell line SW1353 and human OS cell lines MG63, U2OS, HOS, and Saos-2 were grant from Xiangya cell repository. HUVECs, SW1353, MG63, U2OS, and HOS were cultured in Dulbecco modified Eagle medium (Gibco, Burlington, ON) including 5% fetal bovine serum (Gibco, USA) and 1x antibiotics, at 37°C and 5% CO_2_. Saos-2 was cultured in McCoy's 5A (Gibco, USA) with the same additives in the same conditions.

### Real-Time Fluorescence Quantitative Polymerase Chain Reaction (qPCR) Analysis

Cells or tissues were homogenized in TRIzol (CW Biotech, Beijing, China) and total RNA was extracted. The isolated RNA was quantified using Spectrophotometer (NanoDrop, Thermo Fisher, USA) and total RNA (1 μg) was utilized for reverse transcription and cDNA synthesis using designed primers and the Transcriptase III kit (TaKaRa, Japan) following the instructions. Gene expression was gauged using the R-M Mix SYBR ROX (TaKaRa, Japan) and the Applied Biosystems 7300 R-T PCR System (Applied Biosystems). The primer sequences used for *EGFL7* and glyceraldehyde-3-phosphate dehydrogenase (GAPDH) are available in Table 1. The amplification conditions are as follows: 94°C predenaturation 3 min, 94°C denaturation 30 s, 57°C annealing 30 s, 72°C extension 2 min; 72°C prolonging one cycle of 10 min.

**Table 1:** The primers used for Q-PCR

**Name**		**Sequences**
EGFL7	Forward	5′-GCAGCACCTACCGAACCATGTA-3′
	Reverse	5′-TCATCCACATCTGACTGGCAAG-3′
GAPDH	Forward	5′-GGAAGCTTGTCATCAATGGAAATC-3′
	Reverse	5′-TGATGACCCTTTTGGCTCCC-3′

### Western Blot Analysis (WB)

Ice cold pyrolysis buffer (NCM Biotech, Suzhou, China) with protease inhibitor cocktail (NCM Biotech, Suzhou, China) were used to lyse cells. Extracts were centrifuged at 4°C for 12,000 × g/15 min and the supernatant protein concentrations were gauged using the BCA Protein Assay Kit (Beyotime). The same amount of protein (50 g) was separated by SDS polyacrylamide gel electrophoresis and transferred to PVDF membrane. Blocking 1 h with 5% defatted milk in Tris-buffered saline (TBS), the membranes were hatched overnight with primary antibodies [anti-human *EGFL7* antibody (ab50254, Abcam, UK) and anti-human GAPDH antibody (AC002, ABclonal, China)] diluted 1,000-fold in 5% defatted milk in TBS at 4°C. Following primary antibody incubation, the membranes were hatched for 1 h with appropriate HRP-conjugated secondary antibody (AS064, ABclonal, China). The blots were then observed using ECL chromogenic reagent (NCM Biotech, Suzhou, China). Western blot band intensities were semi-quantified by densitometry using Image J software (National Institutes of Health) and normalized to those of the relevant loading control.

### Enzyme-Linked Immunosorbent Assay (ELISA)

The level of EGFL7 protein in cell culture medium was determined using an ELISA kit (CUSABIO, Wuhan, China). The samples were added to a 96-microtiter well-plate pre-coverage with the EGFL7 monoclonal antibody from the kit and hatched at room temperature for 2 h. Samples and biotinylated EGFL7 monoclonal antibody were hatched for 1 h at room temperature. After washing three times with 200× wash buffer from the kit, Streptavidin-HRP was added and hatched for 30 min, then washed three times with 200× wash buffer from the kit in accordance with the instructions. Chromogen TMB substrate buffer was added to induce a colored reaction. The absorbance at 450 nm of each well was determined using a microplate reader (Multiscan MK3, Thermo, USA). All samples were measured in triplicate.

### CCK-8 Assay

Cell Counting Kit-8 (CCK-8) (Beyotime, Hangzhou, China) assay was performed to detect the cytotoxicity of cisplatin to cells, and the IC_50_ (half maximal inhibitory concentration) was calculated. After 24 h of inoculation in 96-well-plate with density of 0.2 × 10^4^, the transfected cells were incubated for 1, 2, 3, 4, and 5 days, respectively. Ten milliliters CCK-8 reagent was added into each pore and incubated for 1 h, then OD value was measured at 450 nm.

### Cisplatin Treatment

The IC_50_ of cisplatin for different OS cell lines was detected using the CCK-8 assay. Cells were inoculated into a 6-well-plate and allowed to grow to ~60% confluence. Then, each cell line was treated with the corresponding IC_50_ concentration of cisplatin for 24 h, after which the cells were harvested.

### Immunohistochemistry (IHC)

The pathological tissues of OS patients and CS patients were fixed in 4% paraformaldehyde and embedded in paraffin. Then the specimens were sliced and analyzed by immunohistochemical (IHC). Specifically, each slide was deparaffinized, dehydrated and antigen repaired by immersion in 0.01 mmol/L sodium citrate buffer (pH 6.0) for 15 min in a microwave oven. The activity of endogenous peroxidase was blocked by incubation with 3% hydrogen peroxide solution at room temperature for 10 min. After rinsing with PBS, the slides were hatched for 1 h at room temperature with primary antibodies. Rabbit anti-human-EGFL7 primary antibodies (19291-1-AP, Proteintech, USA) and rabbit anti-human CD34 antibody (ab81289, Abcam, UK) were used at a dilution of 1:100. The sections were then washed in PBS for three cycle 5 min/times and hatched with anti-rabbit secondary antibody (AS064, ABclonal, China) for 15 min at room temperature. Finally, the signal was developed using 3,3′-diaminobenzidine tetrahydrochloride (DAB), and all slides were stained with hematoxylin. The negative group was hatched with PBS instead of the primary antibody.

### Immunofluorescence (IF) and Immunocytochemistry (ICC)

For IF analysis, each slide was de-paraffinized, dehydrated and antigen repaired. For ICC, cells were only fixed paraformaldehyde. The binding of non-specific antibodies was blocked with 10% donkey serum (1 h at room temperature). Slides were then hatched with primary antibodies (anti-human EGFL7, Proteintech, USA) overnight at 4°C After repeated washes in PBST (the ratio of phosphate buffered saline to tween-20 is 1,000:1), The slide was irradiated with fluorescent labeled secondary antibodies at room temperature for 1 h. The cell nuclei were counterstained with 4′,6-diamidino-2-phenylindole (DAPI) and all the images were obtained under Leica subduction-sp8 imaging microscope.

### Statistical Analysis

All statistical analyses were performed using SPSS 20.0 software package (SPSS Inc., Chicago, IL). The results were expressed by mean ± standard deviation (SD). Furthermore, Statistical differences between the experimental group and the control group were determined by the Student's *t*-test. We considered *P* < 0.05 as statistically significant.

## Results

### Upregulation of *EGFL7* mRNA and Protein in OS Tissues

H&E staining of OS patient tumors showed the classic pathological features of OS and CS (Figure 1A). IHC was used to test the expression level of *EGFL7* protein in OS (before new-adjuvant chemotherapy) and CS tissues. This revealed that EGFL7 protein levels were significantly higher in OS tumor tissues, predominantly localized in the cytoplasm of both vascular endothelial cells and OS tumor cells, compared to the low levels observed in CS tissues (Figures 1C,G). The CD34 expression in the microvessels of OS and CS tissues differed significantly (Figures 1D,H). Western blotting confirmed that CS tissues expressed significantly decreased *EGFL7* protein compared to that of OS tissues (Figures 1B,E). In addition, we analyzed *EGFL7* mRNA levels in OS (before neo-adjuvant chemotherapy) and CS tissues using qPCR, and found that the expression levels of *EGFL7* mRNA in all OS tissues were notably higher compared to the expression in CS tissues (Figure 1F).

### Higher Expression of *EGFL7* mRNA and Protein in OS Cell Lines Compared to ECs and CS Cell Lines

**Figure 2:**
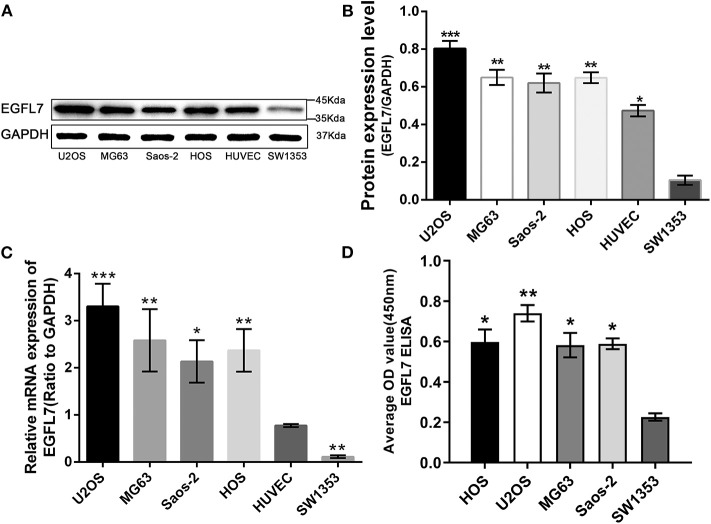
Upregulation of EGFL7 expression in OS cell line compared with ECs and CS cell line. **(A,B)** Western blot results showed that EGFL7 protein was overexpressed in all OS cell lines and HUVECs compared to SW1353. Statistical analysis showed that the expression of EGFL7 in OS cell lines was significantly different from that in SW1353. Similarly, the expression of EGFL7 protein in HUVEC was significantly different from that in SW1353. **(C)** RT-PCR results showed that the transcription level of EGFL7 mRNA in OS cell lines was significantly higher than HUVEC, there are also differences between HUVEC and SW1353, and the difference was all statistically significant. PCR and western blot experiments were all performed in triplicate. **(D)** The ELISA results of EGFL7 in cell culture medium also confirmed that there was indeed a high expression of EGFL7 protein in osteosarcoma cells. *P < 0.05, **P < 0.01, ***P < 0.001.

*EGFL7* protein levels in human OS cells lines U2OS, MG63, Saos2, HOS, human CS cell line SW1353, and HUVECs were analyzed by western blot (Figure 2A). All OS cell lines and HUVECs expressed higher levels of *EGFL7* protein compared to SW1353 (Figure 2B). After analyzing the *EGFL7* mRNA expression levels in human OS cells lines U2OS, MG63, Saos2, HOS, human CS cell line SW1353, and HUVECs, we found that OS cell lines exhibited the highest expression levels of *EGFL7* mRNA, and the level of expression in HUVECs was slightly lower but still significantly higher than that of SW1353 (Figure 2C).

### Expression of *EGFL7* Protein in OS Tumor Cells *in vitro* and *in vivo*

**Figure 3:**
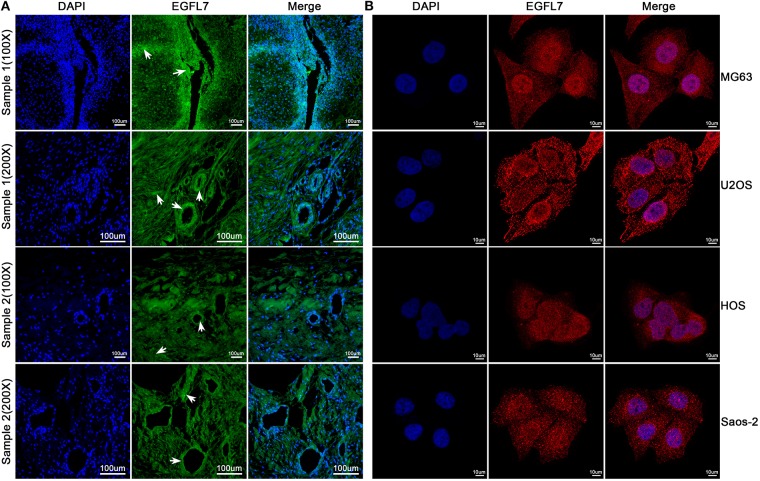
Immunofluorescence technique was used to verify the expression of EGFL7 from histological and cytological aspects, respectively. **(A)** As seen in these representative images, the EGFL7 was labeled by special anti-EGFL7 antibody, and the cell nuclei were stained by DAPI. The merged image shows that green fluorescent signals for EGFL7 protein were expressed in both tumor tissues and vascular endothelial cells, and we have observed this expression from multiple samples and different optical multiples, respectively. White arrows indicate positive areas. **(B)** From the immunofluorescence results of OS cells, the EGFL7 protein was labeled by red fluorescent, the cell nuclei were stained by DAPI. The expression of EGFL7 protein in OS cells is located in the cytoplasm.

The *EGFL7* protein levels in the cell culture medium of human OS cells lines U2OS, MG63, Saos2, HOS, and human CS cell line SW1353 were analyzed by ELISA. The medium of all OS cell lines contained notable levels of EGFL7 protein which was absent in the medium of SW1353, indicating that *EGFL7* protein was expressed by OS cells (Figure 2D). To determine the location of *EGFL7* protein in OS tumor tissues and cells, IF and ICC were used. IF analysis showed that both OS tumor cells and vascular endothelium cells expressed high levels of cytoplasmic *EGFL7* protein (Figure 3A). ICC analysis also revealed that the *EGFL7* protein was localized to the cytoplasm of all four OS cell lines (Figure 3B). These findings indicate that *EGFL7* protein is expressed by OS tumor cells *in vivo* and *in vitro*.

### Deregulation of *EGFL7* mRNA and Protein Expression by Cisplatin in OS Cell Lines

**Figure 4:**
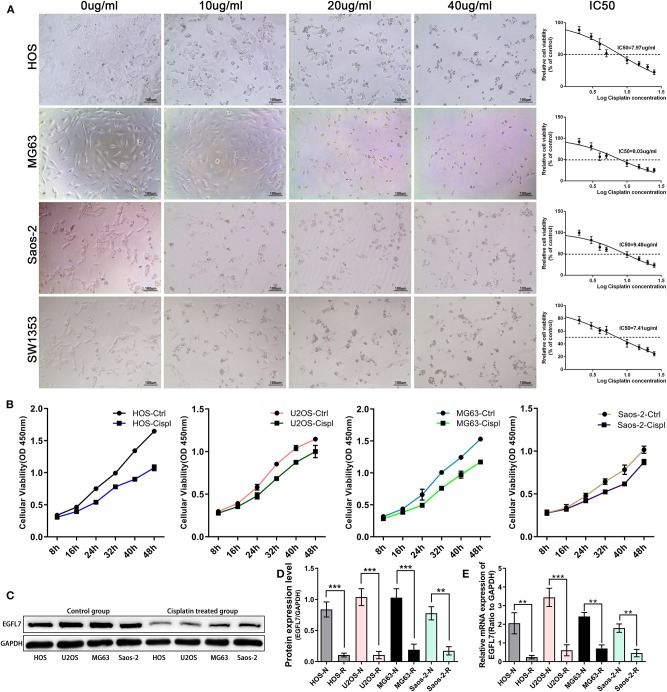
The expression of EGFL7 deregulated in OS cells after cisplatin intervention. **(A)** Intervention of four osteosarcoma cell lines with different gradient concentration of cisplatin. Under light microscope, the number of cells changed significantly with different concentrations of cisplatin. The toxicity of cisplatin was detected by CCK-8 kit and the corresponding IC50 of various cells was calculated. **(B)** The results showed that cisplatin could significantly affect the proliferation rate of tumor cell lines. **(C,D)** Western blot was assessed to compare EGFL7 protein in OS cells before and after Cisplatin intervention, the results showed that the expression of EGFL7 protein deregulated after Cisplatin intervention. Paired T test showed that EGFL7 protein expression in OS cells was significantly deregulated after cisplatin intervention and the difference was statistically significant. **(E)** RT-PCR results showed that the transcription level of EGFL7 mRNA in OS cell lines was significantly deregulated after cisplatin intervention and the difference was statistically significant. PCR and western blot experiments were all performed in triplicate. *P < 0.05, **P < 0.01, ***P < 0.001.

To detect the effect of cisplatin on proliferation, the IC_50_ values of cisplatin-treated OS cell lines U2OS, MG63, Saos2, and HOS were determined through the CCK-8 assay (Figure 4A). Subsequently, four OS cell lines were subjected to cisplatin treatment at the IC_50_ concentration for 24 h. The results showed that cisplatin significantly inhibited the proliferation of osteosarcoma cell lines (Figure 4B). The cells were then treated with the corresponding IC_50_ concentration for 24 h and the resulting mRNA level was analyzed by qPCR. We found that the expression of *EGFL7* mRNA in all cisplatin-treated OS cell lines was notably lower than those in untreated OS cell lines (Figure 4E). Western blotting confirmed the lower expression levels of *EGFL7* protein in cisplatin-treated OS cell lines compared to untreated OS cell lines (Figures 4C,D). Taken together, these results suggest that chemotherapy deregulates the expression of *EGFL7* mRNA and protein in OS cell lines.

### Chemotherapy-Mediated Deregulation of *EGFL7* Expression *in vivo*

**Figure 5:**
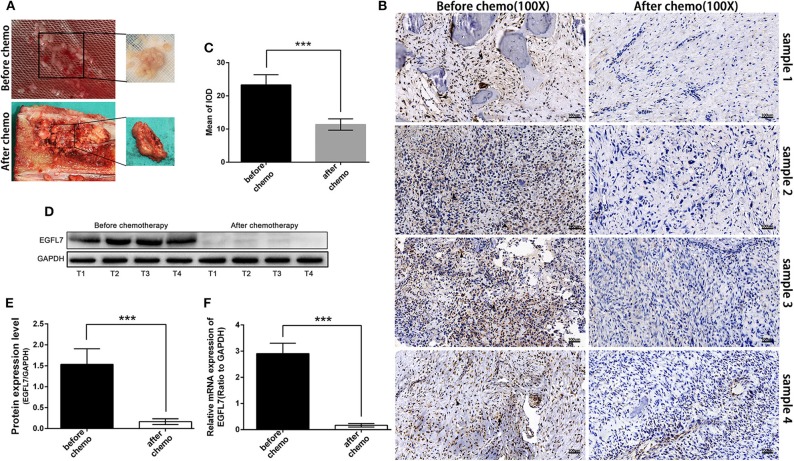
Chemotherapy deregulated expression of EGFL7 in osteosarcoma. **(A)** Samples obtained by biopsy before chemotherapy and by tumor resection after chemotherapy in the same patient. Pre-chemotherapy tissue specimens showed fish-like appearance, while post-chemotherapy tumor tissue showed obvious bone repair changes. **(B,C)** Immunohistochemical staining of the same patient's tumors before and after chemotherapy showed that there was almost no expression of EGFL7 in the tumors after chemotherapy (black arrows represent positive areas). The semi-quantitative statistical analysis based on IHC results shows that there is a significant difference in the expression of EGFL7 between pre-chemotherapy and post-chemotherapy. **(D,E)** Western blot results showed that EGFL7 protein was almost no expression in post-chemotherapy tissue compared with pre-chemotherapy tissue, Student's *t*-test shows that the difference has obvious statistical significance. **(F)** RT-PCR results showed that the transcription level of EGFL7 mRNA in post-chemotherapy tissue was significantly deregulated than that in pre-chemotherapy tissue, and the difference was statistically significant. *P < 0.05, **P < 0.01, ***P < 0.001.

To determine the effect of chemotherapy on EGFL7 expression, we analyzed OS tumor tissues obtained from biopsies before and surgical resection after neo-adjuvant chemotherapy for each patient (matched pairs). The tissues obtained after chemotherapy showed visible residual tumor cells (Figure 5A). IHC showed that *EGFL7* expression was almost completely abolished in OS tissues after chemotherapy compared to untreated OS tissues, which exhibited high *EGFL7* expression levels (Figures 5B,C). Western blotting confirmed lower levels of *EGFL7* protein in OS tissues after chemotherapy compared to that of untreated OS tissues (Figures 5D,E). EGFL7 mRNA expression was also analyzed by qPCR which showed a decrease in *EGFL7* mRNA in OS tissues after chemotherapy compared to untreated OS tumors (Figure 5F). These findings suggest that chemotherapy deregulates the expression of *EGFL7 in vivo*.

## Discussion

OS is the most common malignant primary bone tumor of adolescents and young adults, accounting for 15% of all solid extracranial cancers in children and adolescents. The prognosis of OS is poor due to metastatic relapse and recurrence (15). Chemotherapy, the key procedure for OS treatment, has not improved since the 1980s, mostly due to a lack of understanding of OS pathogenesis (1, 2, 16). As such, there is an urgent need for the identification of specific tumor markers and novel therapeutic targets. *EGFL7* is a secreted protein which contains a signal sequence, an EMI domain at the amino terminus, followed by two EGF-like domains, and a coagulation factor Xa inhibitory domain (3, 17). *EGFL7* is highly expressed during embryonic development; however, it becomes down-regulated in the vascular system at birth (3, 4). The expression of *EGFL7* is then up-regulated again during pathophysiological angiogenesis, gets secreted into the extracellular matrix (ECM), and guides the vascular sprouting process (18, 19). This indicates that *EGFL7* plays an important role in the pathological process of solid tumor growth formation, and thus has potential as a therapeutic target for tumors. Recent studies showed that *EGFL7* is involved in the pathology of certain solid tumors, including breast cancer (7), epithelial ovarian cancer (8), hepatocellular carcinoma (9), colorectal cancer (10), acute myeloid leukemia (11), and malignant glioma (12, 13), However, evidence regarding the role of *EGFL7* in OS is still sparse (14, 20).

This study had two main findings. The first was that *EGFL7* expression by OS tumor cells is upregulated in OS, and this was confirmed using a variety of experimental methods. The results obtained by IHC analysis demonstrated the upregulation of *EGFL7* protein expression in human OS tissues and were further confirmed by western blotting. The expression of *EGFL7* mRNA was also found to be upregulated in OS tissues by qPCR. The higher levels of *EGFL7* mRNA and protein expression were found using OS cell lines, HUVECs, and a CS cell line. ICC and IF analyses showed that *EGFL7* protein was highly expressed in the cytoplasm of OS cell lines and in OS tissues, indicating that the *EGFL7* protein is expressed by OS tumor cells *in vivo* and *in vitro*. High expression levels of *EGFL7* have been previously reported in certain solid tumors; however, the protein was found to be secreted by ECs rather than tumor cells (10, 21). Only a few tumor cells have been found to secrete *EGFL7* (9, 11, 22). Our results showed that the *EGFL7* expression is upregulated by both OS tumor cells and vascular endothelial cells. Interestingly, the amount of *EGFL7* expressed by tumor cells was higher than that expressed by ECs in OS. The other main finding was that chemotherapy deregulates the expression of *EGFL7 in vivo* and *in vitro*. A significant downregulation of *EGFL7* by chemotherapy was confirmed by IHC, qPCR, and WB, in both OS tumor tissues and OS cell lines. The tumor cells still had proliferative capacity after chemotherapy but lost the ability to secrete *EGFL7*. These results suggest that *EGFL7* may be one of the effectors of chemotherapy in OS.

*EGFL7* expression in human cancer needs to be carefully evaluated, as *EGFL7* may play complex roles in cancer, being potentially secreted by tumor cells, ECs, or both. The *EGFL7* gene has an EMI domain at the amino terminus, followed by two EGF-like domains, and is the host gene of miRNA-126 (4, 23). Recent studies have found that *EGFL7* participates in the pathological process of human tumors with a wide range of effects. *EGFL7* attachment to the extracellular matrix, its interaction with integrin αvβ3, or its upregulation of integrin α5β1 increased the motility of ECs, which allowed these cells to move on a sticky underground during vessel remodeling (5, 21, 24). *EGFL7* was also showed to inhibit Notch signaling (23, 25, 26), which is a known key regulator in both cancer development and muscle stem cell activity, by a direct interaction with the Notch family of receptors. *EGFL7* may promote tumor escape from immunity by downregulating the expression of leukocyte adhesion molecules in endothelial cells, thus repressing immune cell extravasation into tumors (6, 27). Moreover, it has been reported that *EGFL7* directly increases tumor cell migration in hepatocellular carcinoma (9) and acute myeloid leukemia (11). Angiogenesis, immune escape, Notch signaling, and the proliferation of tumor cells may be all involved in the pathological process of OS. Our previous findings showed that there was a tumor grade-dependent up-regulation of *EGFL7* in OS tumor tissues, where high *EGFL7* expression levels were associated with a worse prognosis (14). Here, we found that *EGFL7* is upregulated in OS, expressed by both tumor cells and vascular endothelial cells, and that chemotherapy deregulated the expression of *EGFL7 in vivo* and *in vitro*. Thus, it is likely that this protein provides a growth advantage to the tumor cells that express it, having its effect on endothelial cells during angiogenesis.

The fact that *EGFL7* expression is deregulated in OS after chemotherapy and its expression was positively correlated with the prognosis of patients (14). The effect of cisplatin on *EGFL7* expression is temporary or permanent, which has not been confirmed yet. Cisplatin is one of the four first-line drugs in the chemotherapy of osteosarcoma. Previous clinical studies showed that cisplatin can reduce the mortality of patients with osteosarcoma (14). Our cell experiment also found that cisplatin can cause the down-regulation of *EGFL7* expression in tumor cells, but tumor cells are easy to produce drug resistance. Therefore, further cell intervention experiments and animal models are needed to clarify the mechanism of cisplatin on *EGFL7*. OS tumor cells show a much stronger and frequent cytoplasmic signal for *EGFL7* than ECs is not consistent with previous reports of other cancers. *EGFL7* protein was detected in the cytoplasm of human hepatocellular carcinoma cancer cells (9). High expression levels of *EGFL7* transcripts were associated with higher tumor grades in colon carcinoma and glioma, but the *EGFL7* protein was mainly secreted by endothelial cells (10, 21). High expression levels of *EGFL7* were significantly correlated with clinicopathologic factors epithelial ovarian cancer (8) and pancreatic carcinoma (28). However, there was no separate analysis of *EGFL7* expression in endothelial, stromal, or tumor cells in these studies, and since *EGFL7* is highly expressed in endothelial cells, it is probable that these correlations were at least partly due to the high vascularization of advanced tumors, as seen in glioma (21). Some discordance was reported between the mRNA and the protein expression levels of *EGFL7* in acute myeloid leukemia (11), indicating that additional post-transcriptional or post-translational mechanisms could be involved in the regulation of *EGFL7*. These analyses need to be complemented with a histological identification, such as was carried out in this study.

Further research should be carried out on the basis of our study. Some loss function experiment *in vitro* was needed, such as knocking down *EGFL7* by siRNA or lenti-virus, for further verification whether *EGFL7* might be one of the effectors of chemotherapy in OS. Besides, we only used cisplatin to treat cell lines, which indicated cisplatin could down regulate *EGFL7* expression in OS cell line. Some other chemotherapy drugs used in neo-adjuvant chemotherapy such as adriamycin and ifosfamide should be also detected. Moreover, we still are unknown about why *EGFL7* suppressed by chemotherapy or its function in OS progression. We will carry out further research to clarify these issues.

In conclusion, we performed the first thorough study of the expression of *EGFL7* transcription levels and protein localization in human OS tissues and cells, demonstrating that *EGFL7* overexpression in OS could be downregulated by chemotherapy. We have thus shed some light on how OS responds to chemotherapy during decreased angiogenesis, for which *EGFL7* could be a potential therapeutic target.

## Data Availability Statement

The raw data supporting the conclusions of this article will be made available by the authors, without undue reservation, to any qualified researcher.

## Ethics Statement

The studies involving human participants were reviewed and approved by the Research Ethics Committee of Xiangya Hospital. The patients/participants, or in the case of minors, the participants' legal guardian/next of kin provided their written informed consent to participate in this study.

## Author Contributions

HH and WL designed the experiment. QL, YY, and HZ performed the experiment. ZW analyzed the data. QL and WL wrote the manuscript.

### Conflict of Interest

The authors declare that the research was conducted in the absence of any commercial or financial relationships that could be construed as a potential conflict of interest.

## Abbreviations

EGFL7: epidermal growth factor-like domain 7
OS: osteosarcoma
CS: chondrosarcoma
HUVECs: human umbilical vein endothelial cells
IHC: immunohistochemical staining
IF: immunofluorescence
ICC: immunocytochemistry
ECs: endothelial cells
RT-qPCR: real time quantitative polymerase chain reaction
CCK-8: cell counting Kit-8
WB: western blot analysis.
